# Relative lymphocyte count as an indicator of 3-year mortality in elderly people with severe COPD

**DOI:** 10.1186/s12890-018-0685-6

**Published:** 2018-07-13

**Authors:** Domenico Acanfora, Pietro Scicchitano, Mauro Carone, Chiara Acanfora, Giuseppe Piscosquito, Roberto Maestri, Annapaola Zito, Ilaria Dentamaro, Marialaura Longobardi, Gerardo Casucci, Raffaele Antonelli-Incalzi, Marco Matteo Ciccone

**Affiliations:** 1 Maugeri Scientific Clinical Institutes, SpA SB, Institute of Care and Scientific Research, Rehabilitation Institute of TeleseTerme, Benevento, Italy; 2 Section of Cardiovascular Diseases, Department of Emergency and Organ Transplantation, School of Medicine, University of Bari, Bari, Italy; 3 Maugeri Scientific Clinical Institutes, SpA SB, Institute of Care and Scientific Research, Rehabilitation Institute of Montescano, Pavia, Italy; 4 San Francesco Hospital-TeleseTerme, Telese, BN Italy; 5 Institute of Internal Medicine, Chair of Geriatry, Policlinico Gemelli, School of Medicine, Rome, Italy

**Keywords:** Lymphocyte, Mortality, Elderly, COPD

## Abstract

**Background:**

Prognostic stratification of elderly patients with chronic obstructive pulmonary disease (COPD) is difficult due to the wide inter-individual variability in the course of the disease. No marker can exactly stratify the evolution and natural history of COPD patients. Studies have shown that leukocyte count is associated with increased risk of mortality in COPD patients. The aim of this study was to evaluate the possible role of relative lymphocyte count as a risk marker for mortality in elderly patients with COPD.

**Methods and results:**

This is a3-year prospective study. A total of 218patients, mean age 75.2±7 years, with moderate to severe COPD and free from conditions affecting lymphocyte count were enrolled. The population was divided into two groups according to the relative lymphocyte count, with a cut-off of 20%. Eighty-five patients (39%) had a relative lymphocyte count ≤20%. Three-year mortality rates from any cause in patients with relative lymphocyte count ≤ or > 20% were 68 and 51%, respectively (*p =* 0.0012). Survival curve analysis showed higher mortality in patients with relative lymphocyte count ≤20% (*p =* 0.0005). After adjustment for age and sex, the hazard ratio for mortality risk according to lymphocyte count was 1.79 (95% confidence interval [CI]: 1.26–2.57, *p =* 0.0013), even in the analysis limited to the 171 patients without congestive heart failure (1.63; 95% CI: 1.03–2.58, *p* = 0.038).

**Conclusions:**

Low relative lymphocyte count was associated with higher mortality in elderly patients with severe COPD.

**Electronic supplementary material:**

The online version of this article (10.1186/s12890-018-0685-6) contains supplementary material, which is available to authorized users.

## Background

Chronic obstructive pulmonary disease (COPD) is a frequent cause of death in elderly patients.

Older age, reduced gas exchange and airflow obstruction, right atrial overload and ventricular overload and hypertrophy and selected comorbidities are the main negative prognostic indicators as they could account for early death in these patients [1–3].

The prognostic stratification of elderly patients with COPD is difficult due to wide inter-individual variability the course of the disease. No marker can exactly stratify the evolution and natural history of COPD patients. Therefore, efforts are made in order to detect new prognostic markers and to verify whether they substantially add further prognostic value to well-recognized indicators [2, 4, 5]. Incalzi et al. [2] identified electrocardiographic signs (S1S2S3 pattern or right atrial overload (RAO)) as able to predict survival rates in patients with COPD.

The common plasmatic measures and biomarkers such as (procalctionin (PCT), C-reactive protein (CRP), white blood cell count (WBC)) are not able to reproducibly predict mortality in COPD patients [4].

Variables reflecting either inflammation or immune depression emerged as possible prognostic markers in patients with COPD. The increase in leukocyte count in peripheral blood shows a statistical trend towards the prediction of long-term all-cause mortality risk in COPD patients but it did not reach a statistical significance in previous studies [4, 6].

More insights come from the analysis of reduced WBCs and, in particular, lymphocyte count. A low relative lymphocyte count is already known to exert a detrimental prognostic role in the setting of acute myocardial infarction, stable coronary heart disease and congestive heart failure [7–11]. It also has a negative prognostic role in the elderly population [12], and, to some extent, it could be considered as a marker of the stress response [13, 14]. Although a low relative lymphocyte count is a physiological adaptation of the immune system to increasing age, it may account for the frailty of elderly people compared with younger individuals [15–17]. As patients with COPD may show a further decrease in lymphocyte count [18, 19], elderly individuals with COPD may have a combined reduction in lymphocytes due to both age and characteristics of the pulmonary disease.

Despite the established role of low relative lymphocyte count in many diseases, its prognostic value remains to be specifically evaluated in elderly patients with COPD.

The aim of the present study was to investigate the prognostic value of relative lymphocyte count in elderly patients with COPD.

## Methods

### Patients

The study was conducted on elderly patients with COPD who were consecutively admitted to our Institute for Research and Care related to Cardiac and Pulmonary Rehabilitation. The diagnosis of COPD was made according to American Thoracic Society (ATS) criteria [20]. A total of 218 patients (114 men and 104 women), mean age 75.2±7 years, were recruited from October 1, 2011 to March 30, 2012.

Inclusion criteria were: age ≥65 years and presence of severe and very severe COPD, as identified by ATS criteria [20]. Severe COPD was defined in agreement with forced expiratory volume in the 1st second (FEV1) ≤50% of predicted value. Patients were excluded if they were lacking complete blood count within 1 week of study entry, or on the basis of conditions known to affect lymphocyte count [12, 13]: recent coronary revascularization or recent myocardial infarction (within 6 months of entry), long-term disorders of the hemopoietic system, history of malignancy, chemotherapy, or radiation therapy, trauma, surgery, infection and glucocorticoid therapy within 6 weeks of study entry. Patients were also excluded if dyspnoea, phlegm or weakness had worsened in the two weeks prior to admission. This precautionary measure was utilized to limit the risk of enrolling patients with exacerbated COPD.

### Study design

Extensive baseline data were collected for all eligible patients within one week of hospital admission. Patients underwent physical examination, spirometry, chest X-ray, arterial blood gas analysis, electrocardiogram and laboratory tests (including complete blood count); their clinical history was accurately recorded**.** Spirometry was performed with a VMAX 2200 spirometer (Sensor Medics Co., Yorba Linda, USA), meeting the American College of Physicians, American College of Chest Physicians, American Thoracic Society, and European Respiratory Society 2011 recommendations for diagnostic spirometry [20, 21]. Arterial blood gas analysis was performed (during room air breathing) with an ABL 520 Radiometer analyzer (Copenhagen, Denmark).

Patients’ characteristics were reported: age, gender, body mass index, history of smoking (defined as subjects who had regularly smoked at least 5 cigarettes/day during the previous 3 months or who had stopped smoking less than 1 year before admittance into our study), alcohol use, lung function [FEV_1_, forced vital capacity (FVC), both in milliliters and as percent of predicted], arterial blood gases [partial arterial oxygen tension (PaO_2_), partial arterial carbon dioxide tension (PaCO_2_), pH], presence of ischemic heart disease, history of myocardial infarction, congestive heart failure, diabetes, systemic hypertension, cerebro-vascular disease, peripheral vascular disease, use of bronchodilators, mucolytics and aminophylline, heart rate, systolic and diastolic blood pressure. None of the enrolled patients were in long-term oxygen therapy. Furthermore, their PaO2 (mmHg) at rest was 70.4 ± 8 mmHg (range 60–91 mmHg).

Individual clinical history was diagnosed according to the International Classification of Diseases, Ninth Revision, Clinical Modification [22]. Patients were classified as affected by congestive heart failure only on the basis of a concordant diagnosis according to the physician’s clinical judgment, Boston criteria [23] and a clinical diagnostic score which was previously validated on hospitalized patients [24]. We used this conservative diagnostic approach due to the great difficulty in distinguishing chronic cor pulmonare from congestive heart failure.

Baseline clinical evaluation, complete blood count, electrocardiography, lung function, blood gas analysis, and data on patient’s clinical history were collected by a trained and experienced physician.

### Follow-up

The follow-up covered a period of 36 months after hospital discharge. Physicians phoned all the patients at 6, 12, 24 and 36 months after hospital discharge in order to assess the data regarding their health condition. In particular, the primary end-point of the study was “death from any cause”.

Death certificates and hospital records were considered and checked when patients and/or relatives were not available for phone recall. All available data were reviewed by two investigators (DA, MMC) to determine and define the cause of death. If a consensus of opinion could not be reached, the opinion of the senior investigator (RAI) prevailed.

### Laboratory methods

All patients underwent complete blood count evaluation and a leukocyte differential count analysis at baseline. The percentage of lymphocytes was defined as: (total lymphocytes/total leukocytes)× 100. In our laboratories, the normal range of the percentage of lymphocytes is 20 to 50%, as defined by the central 95% range in a separate population of 52 healthy adults [11].

We evaluated the short- and long-term reproducibility of relative lymphocyte count by considering three blood samples from 24 healthy volunteers over a period of 360 days, spaced at 6 ± 1 and 136 ± 67 days.

### Study approval

All procedures were in accordance with the ethical standards of the institutional research committee and with the 1964 Declaration of Helsinki. Informed written consent was obtained from all individual participants included in the study.

### Statistical analysis

Data analysis was performed with SPSS software (SPSS, Chicago, USA). Results were given as means (±standard deviation) for continuous variables or as percentages for dichotomous variables.

Short-term and long-term reproducibility of relative lymphocyte count was assessed by first testing for systematic changes, between baseline and second measurement, and between baseline and the final measurement, respectively. To quantify the reproducibility, we used the standard error of measurement, which was computed as the root mean square error of the 1-way random effects analysis of variance on short-term and long-term paired measurements. From the same 1-way analysis of variance, the intra-class correlation coefficient (an index of reliability of measurements) was derived and remained significantly higher at long-term follow-up (Additional file 1: Table S1).

Differences between groups were assessed by unpaired t-test or Mann-Whitney test for continuous variables with or without normal distribution and homogeneous variance, and by chi square test for dichotomous variables. Kaplan-Meier estimates of the survival functions were plotted for relative lymphocyte count [25]. Univariate and multivariate Cox regression models were used to investigate the association of selected variables with the incidence of death [26].

All hypothesis tests were tested using a significance level of 0.05. All *p*-values were two-sided.

## Results

Relative lymphocyte count showed high short- (standard error of measurement = 2.11) and long-term (standard error of measurement = 1.15) reproducibility. The estimates of intra-class correlation coefficient for the short- (intra-class correlation coefficient = 0.95) and long-term (intra-class correlation coefficient = 0.97) indicated excellent reproducibility for the relative lymphocyte count measurement. These values indicate that obtaining a single sample from a subject is fairly representative of that individual’s relative lymphocyte count over an extended period of time.

In the study sample, 85 patients (39%) had relative lymphocyte counts≤20%.

The study sample included 218 patients with mean age of 75.2 ± 7 years, 52% male. The population sample was divided into two groups according to relative lymphocyte count: group 1 with relative lymphocyte count ≤20%; group 2 with relative lymphocyte count > 20%. About 39% of patients showed a relative lymphocyte count ≤20%.

Demographic, respiratory function and clinical characteristics of the patients in the two groups are shown in Table 1. Groups did not differ according to demographic characteristics or anthropometric status, as expressed by body mass index (BMI). All of the patients were white Caucasic individuals. A lymphocyte count ≤20% was associated with a lower six-minute walking distance and higher basal heart rate (Additional file 2: Figure S1 and Additional file 3: Figure S2). The reproducibility of the white cells count can be observed in Additional file 1: Table S1.

**Table 1:** Clinical characteristics in patients with severe COPD in relation to the relative lymphocyte count at baseline

Variable^a^	Relative lymphocyte count ≤20% (*N* = 85)	Relative lymphocyte count > 20% (*N* = 133)	*p*-value
Sex (M/F)	46/39	68/65	0.67
Age (years)	76±7	74±7	0.06
Bodymass index (BMI)	25.7±5	25.4±4	0.66
History of smoking (n/%)	43 (51%)	72 (54%)	0.61
Alcohol use (n/%)	30 (35%)	48 (36%)	0.90
Lung Function			
FEV_1_ (ml)	762±268	870±268	0.004
FEV_1_ (percent of predicted)	30±9	34±8	0.005
FVC (ml)	1720±656	2038±687	0.001
FVC (percent of predicted)	53±17	62±17	< 0.0001
FEV_1_/FVC (percent)	47±13	44±11	0.16
Arterial Blood Gases			
PaO_2_ (mmHg)	71±9	70±8	0.79
PaCO_2_ (mmHg)	38±5	37±4	0.12
pH	7.40±0.39	7.40±0.41	0.47
Disease (n/%)			
Ischemic heart disease	21 (25%)	49 (37%)	0.027
History of myocardial infarction	20 (24%)	54 (41%)	0.0091
Congestive heart failure	32 (38%)	15 (11%)	0.0001
Corpulmonale	43 (51%)	49 (37%)	0.031
Diabetes	19 (22%)	25 (19%)	0.52
Systemic hypertension	40 (47%)	65 (49%)	0.79
Cerebro-vascular disease	20 (24%)	32 (24%)	0.93
Peripheral vascular disease	11 (13%)	17 (13%)	0.97
Therapy (n/%)			
Bronchodilators	66 (78%)	100 (75%)	0.68
Mucolytics	70 (82%)	101 (76%)	0.26
Aminophylline	53 (62%)	112 (69%)	0.029
Heart rate (bpm)	95±22	82±19	< 0.0001
Systolic blood pressure (mmHg)	139±25	142±20	0.42
Diastolic blood pressure (mmHg)	78±11	81±9	0.031

Differences between groups were assessed by unpaired t-test or Mann-Whitney test for continuous variables having or not both normal distribution and homogeneous variance, and by the chi square test for dichotomous variables

*COPD* chronic obstructive pulmonary disease, *FEV*_*1*_ forced expiratory volume at 1 s, *FVC* forced vital capacity, *FEV*_*1*_*/FVC* Tiffeneau, *PaO*_*2*_ partial arterial oxygen tension, *PaCO*_*2*_ partial arterial carbon dioxide tension, *MI* myocardial infarction. Plus-minus values are means±SD

^a^Bodymass index is expressed as weight (kg)/height^2^ (m) ratio

Lung function indices were significantly lower in patients with relative lymphocyte count ≤20%; the greatest difference was evident in FVC, expressed as a percentage of the predicted value. The FEV1/FVC index did not distinguish between groups because both terms in the ratio had reduced by a comparable extent in the two groups (Table 1).

Ischemic heart disease and previous myocardial infarction were less prevalent in patients with relative lymphocyte count ≤20% (group 1), while congestive heart failure, diabetes and chronic cor pulmonare were more prevalent in group 1. Neither arterial blood gases nor pharmacologic therapy distinguished between the groups, except for a slightly higher use of mucolytics by patients with a lymphocyte count > 20% (group 2).

Only one patient was lost at the 36-months follow-up time (he was known to be alive at 25th month follow up) and was censored. Mean follow-up time was 22 months, with 118 deaths. One- and 3-year survival rates were 67 and 42.2%, respectively. Patients died from acute or chronic respiratory failure [34 (27%)], acute myocardial infarction [31 (25%)], stroke [21 (17%)], cor pulmonare [12 (10%)], respiratory infections [10 (8%)], cancer [9 (7%)], sudden death [4 (3%)], or other causes [5 (4%)].

Low relative lymphocyte count at baseline was associated with an increased incidence of death from any causes [58 (68%) vs. 68 (51%); *p =* 0.0012] (data not shown).

In the Additional file 4: Figure S3 the hazard ratio is shown as a function of relative lymphocyte count threshold. A clear maximum around 20% can be appreciated indicating this value as the optimal cutoff-point.

Table 2 compared causes of early (within 6 months from discharge) and late (after 6 months from discharge) mortality. Worsening of cardiopulmonary failure prevailed as a cause of early mortality, while acute myocardial infarction, stroke and cancer accounted for most late mortality.

**Table 2:** Causes of early (0–6 months from discharge) and late (> 6 months from discharge) mortality

Cause of death	n Early death	n Late death
Progressive pulmonary failure	44	12
Acute myocardial infarction	11	20
Stroke	8	13
Cancer	0	9
Sudden death	1	3
Others	2	3

Pearson’s χ^2^ = 39.57, *p* < 0.001

**Fig. 1:**
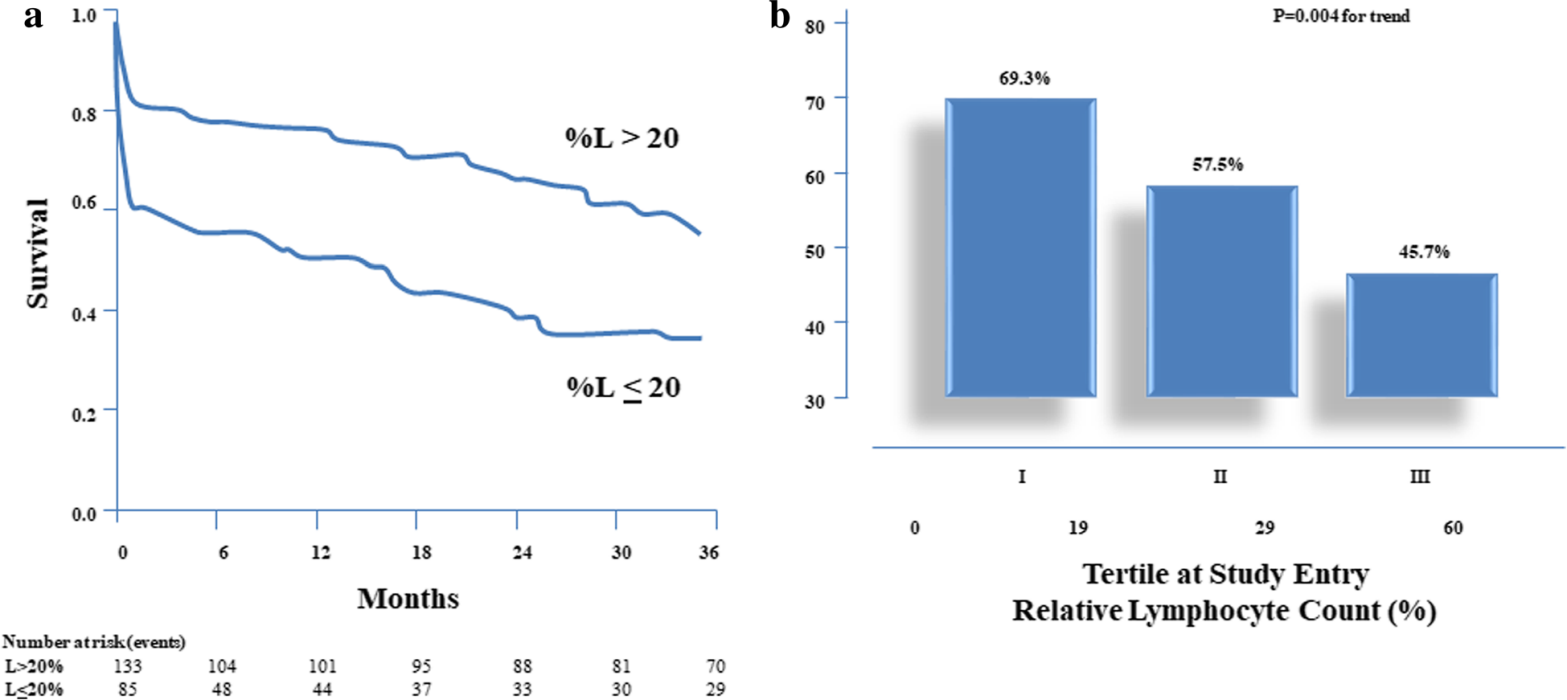
**a**. Survival curves for end-point (death) by relative lymphocyte count. Test statistics for equality of survival distribution for relative lymphocyte count: *p* = 0.0004 by log-rank. %L denotes percentage of lymphocytes. **b**. Three-year survival by tertiles of lymphocyte count at study entry

Kaplan-Meier estimates of the probability of death are shown in Fig. 1a. Patients with relative lymphocyte count ≤20% had worse prognosis, which became evident in the earliest phases of the follow up and remained unchanged for the whole observation period.

The relationship between tertiles of relative lymphocyte count at study entry and 3-year mortality is shown in Fig. 1b. Mortality decreased significantly, from 69.3% in the first tertile to 45.7% in the third.

Table 3 outlines the results of Cox proportional-hazard model comparing mortality in patients with lymphocyte count≤ 20 and > 20%. The age- and sex-adjusted hazard ratio was significantly different from 1.0, suggesting an excess of mortality in patients with lower relative lymphocyte count. The result remained statistically significant even after further adjustment for smoking, body mass index, FEV1 (percent of predicted) and also after adjusting for all variables potentially associated with death.

**Table 3:** Results of Cox proportional hazards model comparing mortality in patients with severe COPD with relative lymphocyte count ≤20%

Variable	Hazard ratio for relative lymphocytes count ≤20% (95% CI)	*p*-value
Adjusted for age and sex	1.81 (1.27–2.59)	0.0012
Adjusted for age, sex, smoking, body mass index, FEV_1_ (percent of predicted)	1.79 (1.22–2.62)	0.003
Adjusted for age, sex, smoking, body mass index, FEV_1_ (percent of predicted), FVC (percent of predicted), FEV_1_/FVC (percent), PaO_2_, PaCO_2_, pH, ischemic heart disease, history of myocardial infarction, congestive heart failure, heart rate, systolic and diastolic blood pressure.	1.56 (1.02–2.38)	0.04

*COPD* chronic obstructive pulmonary disease, *CI* confidence interval, *FEV*_*1*_ forced expiratory volume in one second, *FVC* forced vital capacity, *FEV*_*1*_*/FVC* Tiffeneau, *PaO*_*2*_ partial arterial oxygen tension, *PaCO*_*2*_ partial arterial carbon dioxide tension

Table 4 shows the Cox proportional-hazard model limited to patients without a diagnosis of congestive heart failure. The hazard ratio for lymphocyte count ≤ 20% (group1) remained significant after excluding those with congestive heart failure.

**Table 4:** Results of Cox proportional hazards model comparing mortality in COPD patients free from CHF with relative lymphocyte count ≤20%

Variable	Hazard ratio for relative lymphocytes count ≤20% (95% CI)	*p*-value
Adjusted for age and sex	1.65 (1.04–2.61)	0.034
Adjusted for age, sex, smoking, body mass index, FEV_1_ (percent of predicted), FVC (percent of predicted), FEV_1_/FVC (percent), PaO_2_, PaCO_2_, pH, ischemic heart disease, history of myocardial infarction, heart rate, systolic and diastolic blood pressure.	1.63 (1.03–2.58)	0.038

*COPD* chronic obstructive pulmonary disease, *CHF* congestive heart failure, *CI* confidence interval, *FEV*_*1*_ forced expiratory volume in one second, *FVC* forced vital capacity, *FEV*_*1*_*/FVC* Tiffeneau, *PaO*_*2*_ partial arterial oxygen tension, *PaCO*_*2*_ partial arterial carbon dioxide tension

## Discussion

Relative lymphocyte count was significantly related to survival in elderly patients with moderate to severe COPD. The present findings are in agreement with those of Lehtonen et al. who found that a reduction in both B and T cells could predict mortality in very old people with severe chronic illnesses [15]. The differences in survival at 1 and 3 years between groups with and without relative lymphopenia were similar, with the excess deaths in those with low relative lymphocyte count mostly due to very early events. Figure 1 shows that the difference in survival is early on, and the slopes are the same (i.e. the same relative death rate) from about 6 months afterwards.

Analysis of the causes of death shows that worsening of cardiopulmonary failure was responsible for most of the early deaths, whereas late mortality was related mainly to cardiovascular or cerebrovascular disease and cancer (Table 2). As a sign of impaired immunity, lymphopenia should carry a higher risk of infections, which are the main cause of fatal COPD exacerbations and, thus, should be related mainly to early mortality [5]. The significant difference in both early and late mortality between patients with and without lymphopenia suggests that reduced lymphocyte count qualifies as a comprehensive indicator of health status rather than as a pure immunologic marker. The very high mortality in the six months after discharge from a rehabilitation unit suggests that COPD should not be considered as a stable condition even if clinical judgment is consistent with such a diagnosis. Thus, careful supervision of patients with COPD seems desirable in order promptly to recognize and treat impending cardiopulmonary failure.

There are contradictory results regarding lymphocyte number and function in senescence [15, 27].

Rea et al. demonstrated a 10% decrease in both B and T cell number and percentage of absolute lymphocyte count in elderly subjects [28]. According to B cells, the relative reduction in B cells can provoke alterations in the production of specific antibodies. As B cells can switch immunoglobulins among the different types and in agreement with specific actions toward the pathogenic agents, the reduction in B cell count can negatively affect outcome in elderly patients [29, 30]. Furthermore, T cells can promote the production of cytokines able to enhance the response to pathogens from different actors in the immune system, as well as the switch of immunoglobulins and the promotion of B cell growth and reproduction [28]. All of these data demonstrate that a deficit in either the B or T cell population or both could increase the risk of infection and, thus, morbidity and mortality in elderly patients. Lehtonen et al. suggested that, in very old people, both T and B cell function are significantly reduced and that there are major changes in lymphocyte subsets [15].

The mechanisms leading to the reduction in lymphocyte count and immunological impairment are still far from clear. Aging and COPD are associated with psychological stress [27].

It has been long known that psychological and physiological stresses result in a significant increase in systemic cortisol production [31]. The physiological diurnal variation and pulsatile pattern of secretion are thought to limit the lymphocyte-depleting effect of cortisol. However, elderly people secrete large amounts of cortisol, whose levels may remain elevated for a longer time than in younger adults [32, 33]. Increased cortisol levels can result in a gradual decrease in relative lymphocyte count. Therefore, it may be supposed that a decrease in relative lymphocyte count in elderly patients with COPD, as a consequence of the combined action of age and cortisol pathway, would impair the distribution of white blood cells. Such a theory should be tested in further studies.

The hypothalamus-hypophysis-adrenal axis is a sensitive feedback system for various pathophysiological conditions leading to neurohumoral activation. Several lines of evidence demonstrate that severe COPD is associated with a generalized increase in circulating catecholamines and cytokines [34]. By increasing the production of selected cytokines, mainly of interleukin-6, cathecolamines indirectly stimulate the corticotropin-releasing hormone, which leads to increased secretion of cortisol and a consequent reduction in circulating lymphocytes [35]. Therefore, neurohumoral activation and the immune system demodulation produce a vicious circle that may worsen the prognosis in elderly patients with severe COPD and low relative lymphocyte count. The demonstration that plasma interleukin-6 levels increase with age [36–39] and in patients with COPD [40] supports this interpretation.

Furthermore, activation of the inflammatory systems could be responsible for the cachexia and hypermetabolic state found in some patients with severe COPD [34, 40]. A low relative lymphocyte count can be considered as a surrogate marker for malnutrition in patients with severe COPD, and thus as a negative prognostic factor. Fuenzalida et al. [41] demonstrated a partial recovery in immune system components, and lymphocyte count in particular, in patients with COPD who underwent a dedicated nutritional program. Therefore, the negative prognostic value of low lymphocyte count in elderly patients with COPD could be related to different conditions (cortisol and adrenergic hyperactivation, malnutrition, comorbidities, etc.), which impair the survival of such individuals. Thus, the relative lymphocyte count represents more than a simple marker of immunological deficit.

This study has some limitations, as follows: 1) lymphocyte subpopulations were not measured, and thus the respective prognostic role of deficits of the cellular and humoral immunity could not be assessed; 2) some degree of uncertainty exists in determining the cause of death in this kind of study, and, theoretically, this could affect the interpretation of the relationship between lymphopenia and mortality; 3) orthopnea and fluid retention are not uncommon in severe COPD complicated by hypoxemia and hypercapnia, and could simulate congestive heart failure. However, the stringent criteria used to diagnose congestive heart failure and the results of survival analysis for patients without congestive heart failure support the reliability of the current conclusions. Nevertheless, the proposed prognostic model should be tested in a population with only moderate COPD in order to limit further the potential confounding effect of congestive heart failure.

## Conclusions

Relative lymphopenia has a homogeneous and strong effect on mortality across the whole follow-up period, but the inherent mechanisms remain to be clarified. To our knowledge, this study is the first to demonstrate that relative lymphopenia is associated with a poor prognosis in elderly patients with severe COPD. This finding seems worthy of attention because lymphocyte count is a simple, reproducible, widely available and inexpensive prognostic tool. Further research is needed to verify to which extent lymphopenia improves the prognostic definition based on well-established markers, as well as to assess the relationship linking lymphopenia with indices of neurohormonal activation and inflammation. Clarifying these issues would enable quantification of the weight of this new prognostic marker and, possibly, the design of interventions, e.g. immunological therapy, aimed at its correction.

## Additional files

Additional file 1: **Table S1.** Evaluation of the reproducibility of measurements during the follow-up period. (DOCX 17 kb)

Additional file 2: **Figure S1.** Correlation between relative lymphocyte count and 6-min walking test. (TIF 100 kb)

Additional file 3: **Figure S2.** Correlation between relative lymphocyte count and heart rate. (TIF 126 kb)

Additional file 4: **Figure S3.** Hazard ratio as a function of relative lymphocyte count threshold. (TIF 151 kb)

## Abbreviations

ATS: American Thoracic Society
BMI: Body mass index
COPD: Chronic obstructive pulmonary disease
FEV1: Forced expiratory volume in the 1st second
FVC: Forced vital capacity
PaCO2: Partial arterial carbon dioxide tension
PaO2: Partial arterial oxygen tension
