# Supplementary material for: Relative lymphocyte count as an indicator of 3-year mortality in elderly people with severe COPD
Source: BMC Pulm Med. 2018 Jul 13;18:116. doi: 10.1186/s12890-018-0685-6 (PMC6045885; doi:10.1186/s12890-018-0685-6)
Supplement: Supplementary file 1 — Table S1. Evaluation of the reproducibility of measurements during the follow-up period. (DOCX 17 kb) [file 12890_2018_685_MOESM1_ESM.docx]

**Table S1.** Evaluation of the reproducibility of measurements during the follow-up period.

|  | **1° Study** | **2° Study** | **3° Study** |  |
| --- | --- | --- | --- | --- |
| **Total white blood cells (n/ml)** | 7333±2203 | 7400±2237 | 7233±2171 |  |
| **Neutrophils (%)** | 63±5.8 | 62.7±7.2 | 60.2±6.3 |  |
| **Eosinophils (%)** | 2.4±1.3 | 3.1±1.4 | 3.8±3.1 |  |
| **Basophils (%)** | NA | NA | NA |  |
| **Lymphocytes (%)** | 32.2±5.7 | 32±7.6 | 32.9±5.3 |  |
| **Monocytes (%)** | 2±0.96 | 2.2±0.64 | 3.1±1.4 |  |
|  | **F**  **(1s-2s)** | **P**  **(1s-2s)** | **SEM**  **(1s-2s)** | **ICC**  **(1s-2s)** |
| **Total white blood cells (n/ml)** | 2.167 | 0.153 | 166.4 | 0.99 |
| **Neutrophils (%)** | 1.86 | 0.185 | 1.80 | 0.95 |
| **Eosinophils (%)** | 4.33 | 0.047 | 1.17 | 0.23 |
| **Basophils (%)** | NA | NA | NA | NA |
| **Lymphocytes (%)** | 0.153 | 0.699 | 2.09 | 0.94 |
| **Monocytes (%)** | 3.25 | 0.083 | 0.5 | 0.75 |
|  | **F**  **(1s-3s)** | **P**  **(1s-3s)** | **SEM**  **(1s-3s)** | **ICC**  **(1s-3s)** |
| **Total white blood cells (n/ml)** | 1.75 | 0.198 | 278 | 0.98 |
| **Neutrophils (%)** | 31.07 | 0.000 | 2.05 | 0.89 |
| **Eosinophils (%)** | 5.78 | 0.024 | 2.04 | 0.374 |
| **Basophils (%)** | NA | NA | NA | NA |
| **Lymphocytes (%)** | 4.33 | 0.047 | 1.17 | 0.96 |
| **Monocytes (%)** | 11.6 | 0.002 | 1.2 | 0.0 |

1s-2s: time difference between 1° and 2° study (equal to 5±1 days, range 3-7 days)

1s-3s: time difference between 1° and 3° study (equal to 336±251 days, range 52-814 days)

ICC: intra-class correlation

SEM: standard error of the mean
