# Supplementary figures and images for: Relative lymphocyte count as an indicator of 3-year mortality in elderly people with severe COPD
Source: BMC Pulm Med. 2018 Jul 13;18:116. doi: 10.1186/s12890-018-0685-6 (PMC6045885; doi:10.1186/s12890-018-0685-6)

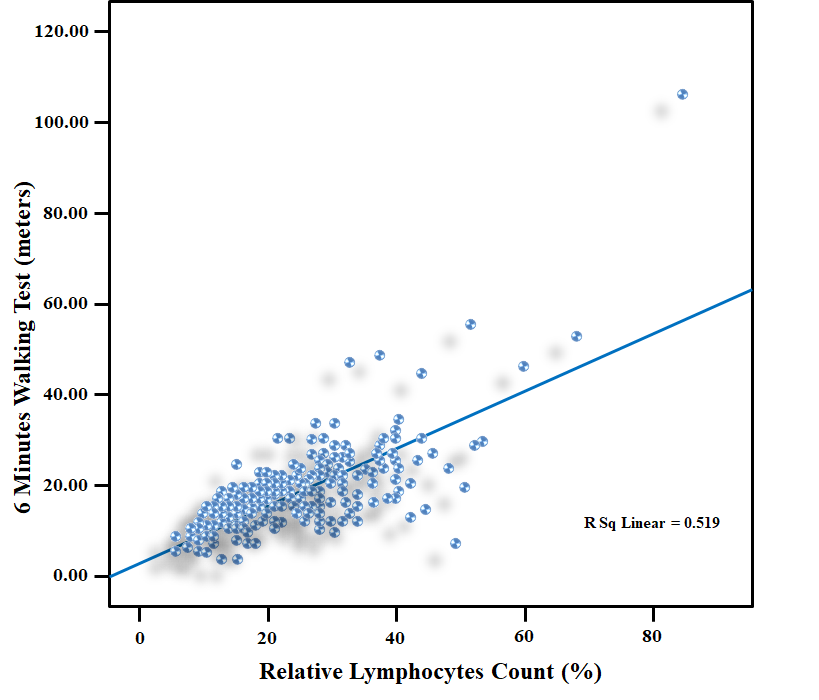

Supplement: Supplementary file 2 — Figure S1. Correlation between relative lymphocyte count and 6-min walking test. (TIF 100 kb) [file 12890_2018_685_MOESM2_ESM.tif]

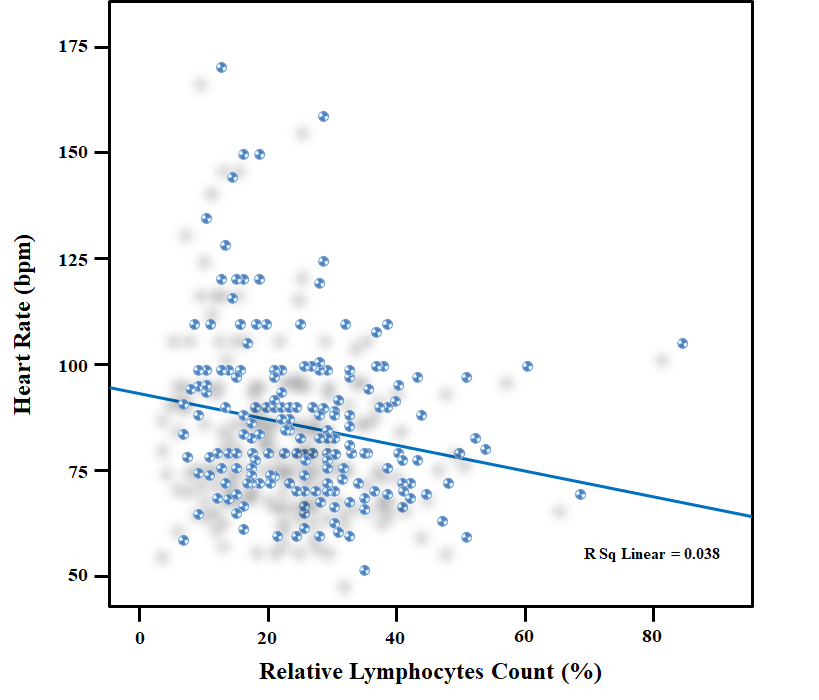

Supplement: Supplementary file 3 — Figure S2. Correlation between relative lymphocyte count and heart rate. (TIF 126 kb) [file 12890_2018_685_MOESM3_ESM.tif]

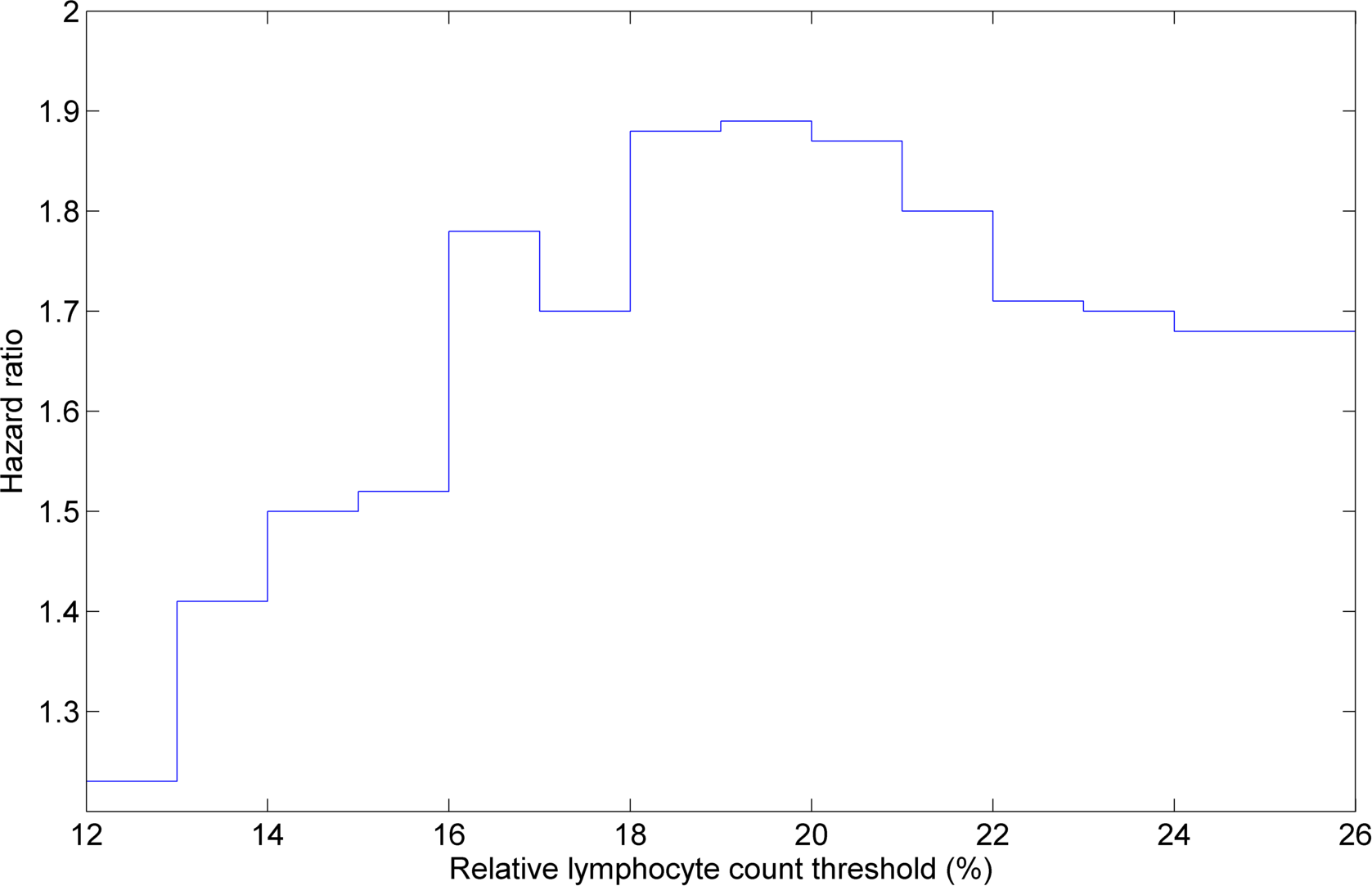

Supplement: Supplementary file 4 — Figure S3. Hazard ratio as a function of relative lymphocyte count threshold. (TIF 151 kb) [file 12890_2018_685_MOESM4_ESM.tif]
